# Implementation of 3D Printing Technology in the Field of Prosthetics: Past, Present, and Future

**DOI:** 10.3390/ijerph16091641

**Published:** 2019-05-10

**Authors:** Albert Manero, Peter Smith, John Sparkman, Matt Dombrowski, Dominique Courbin, Anna Kester, Isaac Womack, Albert Chi

**Affiliations:** 1 Limbitless Solutions, University of Central Florida, 4217 E Plaza Drive, Orlando, FL 32816, USA; peter.smith@limbitless-solutions.org (P.S.); john.sparkman@limbitless-solutions.org (J.S.); MattD@limbitless-solutions.org (M.D.); dominique@limbitless-solutions.org (D.C.); a.kester@limbitless-solutions.org (A.K.); 2 Division of Trauma, Critical Care & Acute Care Surgery Department of Surgery, Oregon Health and Science University, 3181 SW Sam Jackson Park Rd, Portland, OR 97239, USA; womack.isaac@gmail.com (I.W.); chia@ohsu.edu (A.C.)

**Keywords:** prosthetics, 3D printing, cooperative expression, gamification

## Abstract

There is an interesting and long history of prostheses designed for those with upper-limb difference, and yet issues still persist that have not yet been solved. Prosthesis needs for children are particularly complex, due in part to their growth rates. Access to a device can have a significant impact on a child’s psychosocial development. Often, devices supporting both cosmetic form and user function are not accessible to children due to high costs, insurance policies, medical availability, and their perceived durability and complexity of control. These challenges have encouraged a grassroots effort globally to offer a viable solution for the millions of people living with limb difference around the world. The innovative application of 3D printing for customizable and user-specific hardware has led to open-source Do It Yourself “DIY” production of assistive devices, having an incredible impact globally for families with little recourse. This paper examines new research and development of prostheses by the maker community and nonprofit organizations, as well as a novel case study exploring the development of technology and the training methods available. These design efforts are discussed further in the context of the medical regulatory framework in the United States and highlight new associated clinical studies designed to measure the quality of life impact of such devices.

## 1. Introduction

Prosthesis design can be dated back to the ancient Egyptian and Roman empires and has continued to develop across the world throughout the course of history [1,2]. In the late 1800s, John Hanger’s prosthesis, the Hanger Limb, was developed in response to the American Civil War [3], ushering prosthesis design into the modern era. Medical advancements since the invention of the Hanger Limb have significantly reduced limb loss due to traumatic events [4,5]. In 2005, an estimated 1.6 million people in the United States had a limb difference [6], and approximately 541,000 individuals had some level of upper-limb loss [6]. Based on current projections, this value may double by 2050 [6]. Trauma remains the most significant cause of upper-limb amputation, predominantly for males [7], though the subsection of dysvascular-driven adult amputations is rapidly growing. Global monitoring for congenital limb loss is reported annually by the International Clearinghouse for Birth Defects Surveillance and Research Annual Report [8]. It is reported that congenital and pediatric amputations account for a significant population [9,10,11] of overall limb difference.

In the United States, more than 32,500 children have experienced a major pediatric amputation [10]. The Centers for Disease Control and Prevention highlights an estimate that approximately 1500 children are born with upper-limb reductions each year, or approximately 4 of 10,000 live births [12]. Internationally, limb reductions vary from 7.8/10,000 [9,13] (France) to 13/10,000 [14] (Finland), 21.1/10,000 [14] (Netherlands), and 30.4/10,000 [14] (Scotland). Due to the variety of complexities limiting both access and affinities to devices, usage rates by amputees with a prosthesis are still limited.

Substantial percentages of people with congenital limb loss or acquired limb loss choose not to use a device, despite having access to one [15]. Usage rates have been reported for upper-limb devices between 37% [16,17] and 56% [18] among individuals with upper-limb loss. Lower-limb devices are often viewed as more of a necessity than upper-limb devices and have usage rates that vary in literature between 49% [19] and 95% [20]. This difference is particularly expressed among children with transverse upper-limb amputations [21], where usage rates fall between 44% and 66% [22,23,24].

Low usage rates of upper-limb prostheses may result from a lack of aesthetic design, weight, availability of insurance and health care, and high costs [25]. Additionally, device acceptance is complex at the user, provider, parental, and insurance levels. The combination of form and function in the design of prostheses has emerged to provide a higher degree of functionality patterned after the organic 21 degree of freedom human hand [2,26]. Much of the design efforts have been prioritizing achieving a high degree of realism in comparison to the organic analog. Graham Pullin proposed in his book, *Design Meets Disability* [27] that prostheses should not be limited to functional design and that duality should exist between aesthetics and functionality.

Limited research has focused on the benefits of improved aesthetics of prosthetic limbs [28]. Research has found that those with limb difference may have lower self-esteem and higher concern of others’ negative overall perception of their body image [29]. Psychosocial development effects and quality of life considerations for this demographic are still being understood. Research has found that those with limb difference can have lower self-esteem and an overall negative perception of their body image. Donovan et al. [28] highlighted the use of a prosthesis improved social engagement and confidence in those with limb difference. This study did not focus on the visual treatment or appearance of the limbs. Murray [30,31] approached their studies from the prosthesis user’s perspective and found that prostheses use improved the psychoemotional health of those who wore them. Additional research investigating both the functional benefits of prostheses and the role of aesthetic design on the user’s psychosocial development could lead to improved design considerations.

## 2. Contemporary Issues

3D printing is becoming an integral part of upper-limb prosthesis, resulting in response to several tangible issues, including reduced access to conventional prostheses in a timely manner and, in some cases, restricted access. This review focuses on the history of 3D printed prostheses, the populations they support, and the concerns that have driven the work. This is followed by a discussion on the path forward to link the current outcomes into the medical system.

### 2.1. Prosthetic Limb Abandonment

Expectations and daily goals for patients using their prosthetic device have been shown to differ depending on the style of the device the patient receives [32]. An investigation found for device users that the relative importance of many factors, including comfort, vary based on the perception of the device [32]. It can be surmised that a device with increased hand articulation will change the user’s expectation of needed comfort, decreasing its relative weighted importance to the user. The promise of increased control establishes an expectation and level of importance for robust performance. It has also been seen that the factors that contribute to rejections change with the type of amputation method, gender, and age [25]. Congenital limb loss patients are more likely to reject and forgo using a device as an adult, while females with acquired limb loss were more likely to reject devices than their male counterparts [25]. Prosthesis abandonment is a major issue in all populations and can be caused by many reasons. In the adult population, sensory feedback, appearance, function, control, comfort, and durability were all cited as key areas in need of study concerning prosthesis design and acceptance [25].

Biddiss et al. [33] reviewed over 200 research articles and found that pediatric rejection rates ranged from 38% for passive devices, 45% for body-powered devices, and 32% for electric devices. For adults, rejection rates range from 39% for passive devices, 26% for body-powered devices, and 23% for electric devices. Myoelectric devices were not prevalently seen in long term follow-up [34]. The factors driving rejection rates for devices necessitate a conversation of how new approaches can be taken to improve user affinities and outcomes.

### 2.2. Appearance

Those with limb difference can be ostracized due to their perceived impairments. The benefits of having a prosthetic limb can help eliminate some of that stigma. The additional inclusion of art and design in prosthesis development can further empower those involved. Goffman [35] theorized that stigma or rejection by others lead those with disabilities to try to compensate by adopting practices that would hide the disability. This response may be seen in those with limb difference adopting behaviors like holding their different limb behind them for pictures, diminishing the ability for viewers of the picture to notice.

Prostheses for children have often comprised a body-powered hook or a skin tone colored passive device. Even at the current time of publication, passive or body-powered devices with a hook, which may have a silicone glove for aesthetics, are still a common course of care [25,32]. Current trends in prostheses are to push normalization and reduce the level of stigma a user may encounter. Frank [36] has shown that early prosthesis users have found empowerment through various methods. These positive interactions are not limited to the user’s social engagement but also allow the individual to develop their personal acceptance of their limb difference. This development is a complex process [29]. With modern materials, the ability to simulate a natural limb look is becoming more feasible. Huang et al. [37] proposed a LivingSkin™ silicone elastomer gloving material and novel motors to a more realistic look without increasing weight. These devices aim to project a natural appearance; as seen in other fields, art can give those with disabilities ways to express themselves while also increasing their self-esteem [38].

Our research team has theorized that the blend of aesthetic design for functional prostheses, including those that diverge from traditional human form, may be able to support the development of positive social identities and interactions. This will be a point of investigation for the investigators as the research progresses.

### 2.3. Function and Control

Three methods for control of modern bionics include [39]: *(i)* Body-powered through cable extension or contraction, *(ii)* button press [40,41], and *(iii)* electromyography (EMG). All three methods provide unique experiences that users may prefer for use in daily life. Body-powered devices require the complete movement of a different section of the body to move a mechanical section of the hand, claw, or fingers. This additional functionality goes beyond the cosmetic and looks to address specific tasks the individual will encounter in daily life. The selection of a prosthesis is often based off a patient’s particular needs, experience, and functional requirements [42]. Often, in the case of children, their participation in the selection process can be limited. Long duration studies comparing patient outcomes of children fit with devices to those without have not been well reported [43].

Body-powered devices are more predominantly prescribed in the United States and have, in many cases, been viewed as a more robust option than myoelectric devices [25,32,34]. Body-powered devices provide users with a physical sensation feedback, while myoelectric devices may only provide visual feedback [44]. Because of this and further challenges related to robustness, training, and technical limitations, such as overall system weight [25,32], many professionals and users are reported to prefer body-powered devices. When using skin movement or button-press techniques, these control schemes require the use of an essential part of the patient’s body to control this function, which may result in a high degree of false positives, giving the user a bad experience.

Electromyography uses the measurement observation of the resulting electrical potential of a muscle during contraction [45]. This method has stood out due to its many benefits, including small form factor, reliability, and stability. An EMG sensor that has an amplification and rectification circuit to condition the signal and an integrated band pass filter to remove unwanted artifacts and noise can capture the intentionality of the user through a muscle contraction. This filtered signal is able to correlate the intensity of a patient’s contracted muscle, which can be used in actuating the electromechanical hand’s function states. Many prosthetic systems use a group of sensors placed on a set of local muscle groups to capture a set of signals for engagement, or calibrate the resulting signal set derived from various organic (amputated) limb motions [46,47].

## 3. A New Approach to Prosthetic Limb Design

**Figure 1:**
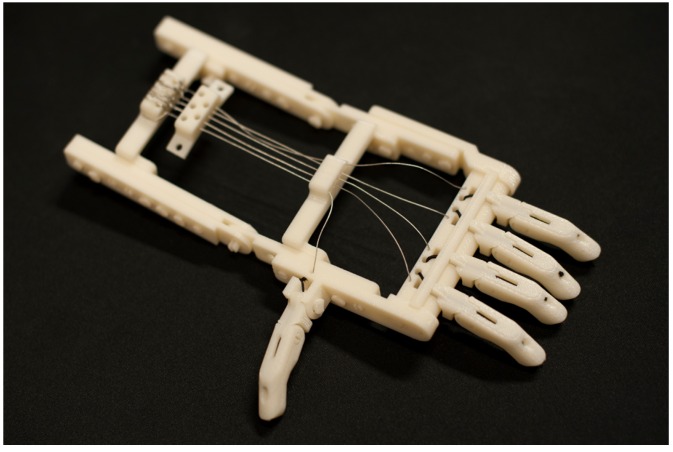
The Robohand assistive device, first made available for 3D printing globally via Thingiverse. Image from the Food and Drug Administration https://www.flickr.com/photos/fdaphotos/9564033498.

Being able to digitally share 3D design models through the internet has led to a growing maker community globally. A carpentry accident in 2011 led to a global collaboration in an effort to regain some of the carpenters’ lost dexterity due to the loss of several fingers [48]. The carpenter, Richard Van As, enlisted the help of a mechanical special effects artist Ivan Owen. This collaboration led to the world’s first 3D-printed upper-extremity prosthesis device in 2012, and the designs were uploaded as an open-source format for the global maker community to reproduce and evaluate. This body-powered device utilized the wrist-flexion of the residual limb to activate uniform contraction of the phalanges and is featured in Figure 1.

### 3.1. The Rise of 3D Printed Prosthetic Arms

The availability of the designs led by both the Robohand and the designer Ivan Owen had a lasting effect globally on the potential application for a variety of accessibility technologies. This inspired researchers and maker enthusiasts to contact the designers wanting to have a similar impact. Upon seeing the effectiveness and impact of collaborative design and production, several maker communities and nonprofit organizations were developed to support local access in their communities, including: e-NABLE (http://enablingthefuture.org/), Enable Community Foundation (http://next2.e-nable.me/), Robohand (http://robobeast.co.za/rich-van-as/), and Limbitless Solutions (https://limbitless-solutions.org/). These groups have included both local at home designers as well as research groups based at various universities, including: Rochester Institute of Technology (RIT), Creighton University, University of Central Florida (UCF), and the University of Washington at Bothell. Additive manufacturing techniques have utilized everything from home-built kit 3D printers to industry-grade machines. While much of the work has been done for body-powered devices, some groups have pushed the research on electromyographically actuated devices to accommodate for higher degrees of limb loss via biosensing and electromechanical motors [49].

Custom sizing, designed specific to the end-user through either volumetric scaling or more precise parametric tailoring, allowed for rapid production and iteration. While many printers now allow for a variety of colors of filament materials, the same base 3D model could be constructed with a user-specified color scheme. This has provided additional affinities and involvement for participatory design. In 2014, a conference for “Prosthetists Meet 3D Printers” was held at Johns Hopkins Hospital, bringing together the maker community and medical professionals, including surgeons, prosthetists, and therapists, to discuss the use of 3D printing for improving access and quality of care [50]. This conference and the e-NABLE web platform were coordinated by a team, including Jen Owen and Jon Schull. The conference brought together a significant amount of limb-different individuals, designers, and medical professionals.

**Figure 2:**
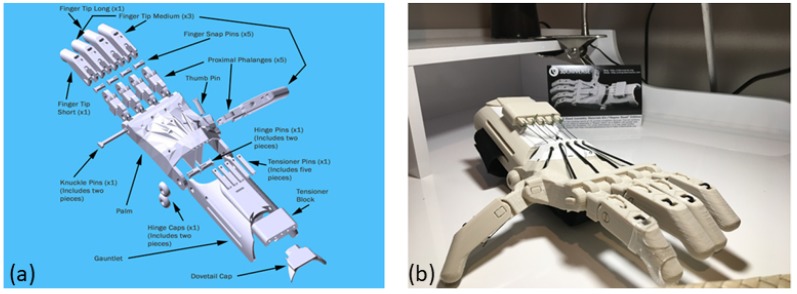
The Raptor reloaded hand by Enable available for download via Thingiverse. (**a**) Exploded view of design and user assembly methods. (**b**) Completed assembly of device. https://www.thingiverse.com/thing:476403.

Collaborative design efforts, many utilizing cloud-based real-time design software such as Autodesk Fusion 360, allowed for group support and advances in the functionality, robustness, and a user-driven feedback opportunity. The Enable network developed substantial advances, which were made available through the Thingiverse.com website’s open-source, with attribution, repository. These design schematics and an image of an example printed and assembled part were made available (https://www.thingiverse.com/thing:476403) and are presented in Figure 2. These devices have reached new heights of accessibility for children all over the globe, made possible due to the availability of open-source customizable designs and new 3D printers used in schools, libraries, and even residences [51,52].

This type of global support has allowed for an accelerated prototyping phase utilizing such collaborative design mindsets. A repository or “family tree” of how the designs and global chapters have progressed is visually available on the https://e-NABLE.org website, including a full visualization at https://kumu.io/jonschull/devices.

**Figure 3:**
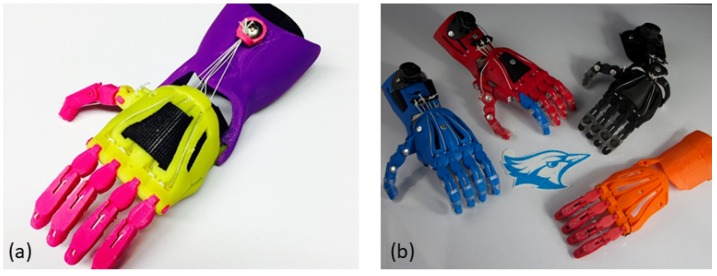
The Cyborg Beast by Creighton University’s Jorge M. Zuniga and available on Thingiverse https://www.thingiverse.com/thing:261462. (**a**) Personalized assembled device. (**b**) A group of assembled hands featuring different cosmetic treatments.

As the maker movement has continued to propagate, there has been integration with the university research environment. Some research groups have sought to standardize production methodologies and establish best practices through data-driven analytics. One example is the work of Jorge M. Zuniga, established during his time at Creighton University (at time of this paper’s publication, at the University of Nebraska). Their work [53,54,55] has pushed the design efforts and field regarding the implementation of additive manufacturing to accelerate bio-medical research and its translation to the medical environment. Their design of the Cyborg Beast hand, a wrist powered design, built on the prior work and improved integration and assembly challenges, has seen significant implementation for children with limb differences and is pictured in Figure 3.

One of the founders and curators of the e-NABLE movement, Jon Schull, and his research team developed significant contributions to the field at RIT, including new body-powered forearms and hands actuated by elbow or wrist movement. This has led to substantial developments for introducing new educational techniques incorporating project-based learning [56] utilizing 3D printers and global design networks [57].

### 3.2. Appearance-Cooperative Expression

Design work using 3D printers has allowed for a higher degree of individual customization of devices. As the collaborative minded network of developers has grown, the role of user-driven design points has been of significant emphasis. In an effort to improve affinities to bionic designs, participation by the end-user has been prioritized. This effort, applied by our research team to prosthesis design, is described as “cooperative expression” and is built on the methodology of participatory design and its strategies, such as cooperative inquiry.

Participatory design represents a field of research, distinguished by a variety of methods, investigating the role of direct user participation with designers [58]. While this was originally discussed for computer-based systems in the workplace, it can also be applied for learning from children and their perspectives while in the development of low-tech design prototypes [59]. In the design process, specific inquiries in the brainstorming methods have become known as cooperative inquiry [59,60]. Druin et al. [59] categorized three dimensions related to children as design partners to be considered, including: *(i)* The child’s relationship to the participating adults, *(ii)* their relationship to the technology, and *(iii)* the goals for the inquiry. Cooperative inquiry, when applied as a method of developing technology, is flexible in construct. Foss et al. [61,62,63] applied the technique to study the role of children with special learning needs and adults as partners in the design process of software while empowering children to customize their experience [62,63]. Their study using a cooperative inquiry approach found reports of emotional engagement in children when the method was applied. Ultimately, this higher engagement is speculated to develop more ownership of the project for the children [62].

A new modified participatory design approach, entitled cooperative expression, is now being applied to visual aesthetic treatment in an effort to improve affinities to the bionic limbs. Our design team’s efforts have taken this participatory and cooperative approach to support the customization of the aesthetics for 3D-printed bionic limbs. Recipients of the bionics have the ability to artistically customize their interchangeable sleeves using an interactive website. Various 3D designs can be compared, selected, and further personalized with customizing color and effect regions. Artists support the initial creation of the aesthetic scaffolding, such as the design of color pallets and the discretization of the zones for customization. This scaffolding is designed to provide an initial framework to minimize selection fatigue and maximize the participants’ ability to explore artistic designs potentially outside their reference frame.

**Figure 4:**
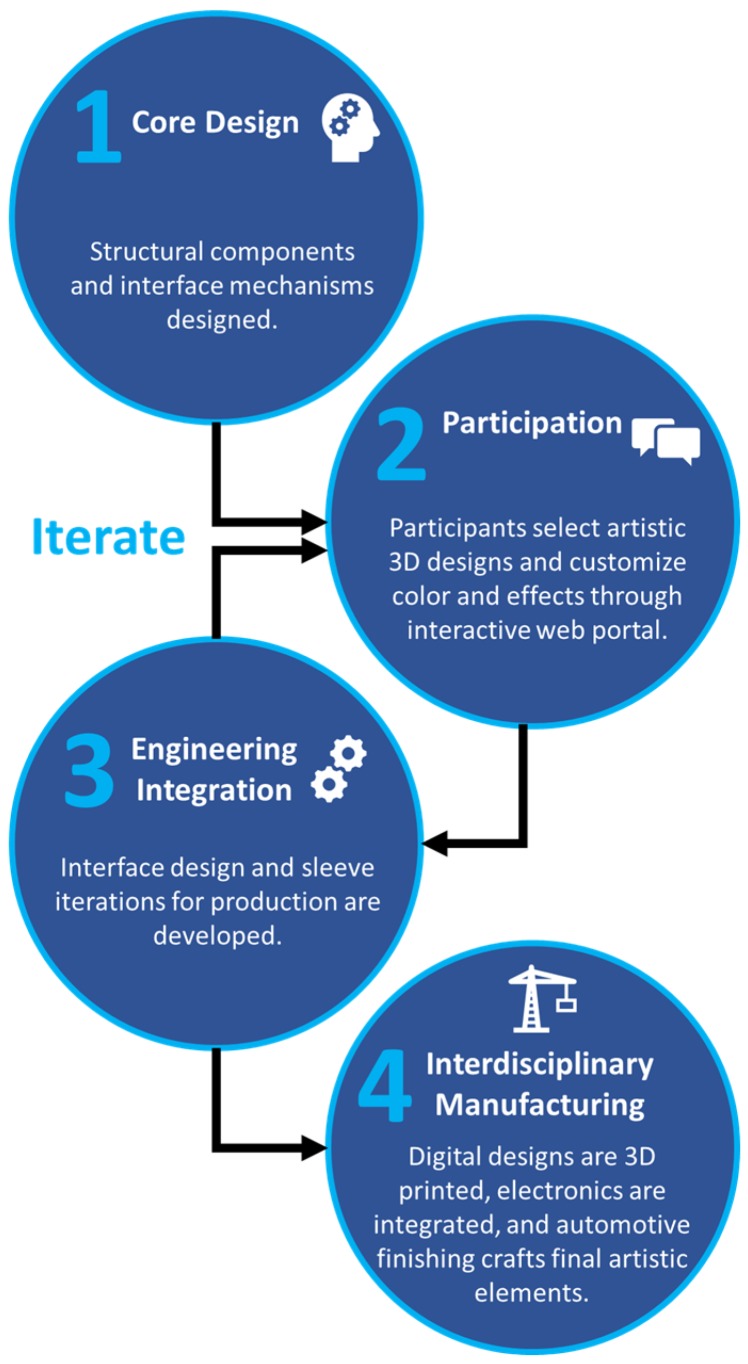
Overview of design process and methodology from design generation, user participation, and interdisciplinary manufacturing.

**Figure 5:**
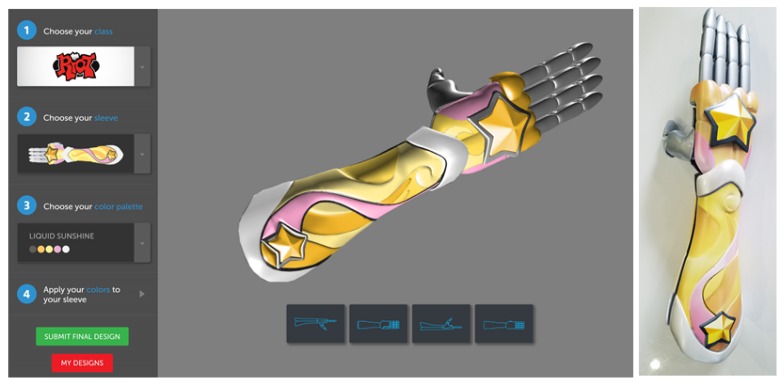
(**Left**) Example interactive web page for children to customize color and effect regions during the design process, and how user participation can be translated to (**Right**) the final design with artistic input from art team and production teams. Sleeve design made in partnership with Riot Games.

This unique customization design process and methodology highlighted in Figure 4 looks to integrate the end-user from start to finish in the design process. The structural and mechatronic components of the arm have been standardized for the digital designers to create a digital 3D representation of the artistic shell. An interactive web portal allows the user to customize colors and effects and regions of the sleeve, allowing expressive visualization of the final design. In some cases, modifications are made to the artistic design visualized on the user portal to support the human–machine interface. Production of the aesthetic sleeves uses an interdisciplinary process including: 3D printing, surface preparation and priming, automotive finishing techniques, and painting. During the painting process, artists capture the effects and colors selected through the user portal and deepen the visual effect. The full system is then validated and prepared for fitting to the participant. This system allows participants to be actively involved with their arm before production or fitting, and an example interaction is presented in Figure 5. This early interaction is anticipated to establish an emotional connection to the limb before the participant is fitted.

**Figure 6:**
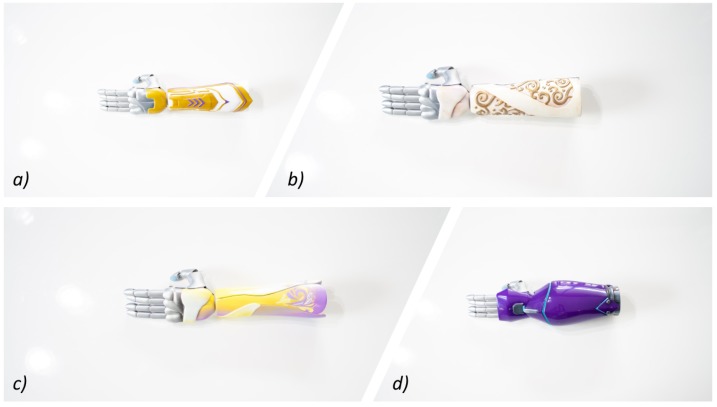
3D-printed electromyographic actuated limb device with interchangeable artistic covers from Limbitless Solutions at the University of Central Florida. (**a**) Warrior class, (**b**) Ethereal class, (**c**) Serenity class, and (**d**) Shadow class.

Part of the design process offers the ability for different categories or “empowerment classes” of interchangeable aesthetic sleeves. These classes are broken down into four individual groups, *Warrior, Shadow, Ethereal, and Serenity*. These classes are designed to represent different personalities linked to emotional affinities. Artists create these inspired 3D models to connect with these personalities, and in some cases, external artists representing characters have added designs to the catalog. Examples of the “empowerment classes” are presented in Figure 6. This variety coupled with the interchangeable options allows the child to have more freedom over their expression; this should improve development of affinity to the device, lower social stigma factors, and support a longer-term engagement, thereby improving user performance. An evaluation of the role of the device on psychosocial development and stigma factors will be conducted in future research.

### 3.3. Function–Electromyography

Antfolk et al. [44] found that a 16-sensor EMG was capable of predicting participants’ desired control with 86% accuracy after a two-day training session. This system was used with a 25-year-old male transradial amputee. The result was produced by the system learning how the user’s EMG input should be interpreted, based on movements performed similarly to how they would have been prior to the amputation [44]. Much of the increased complexity arises from the use of multiple EMG inputs mapping to multiple computer-controlled outputs. Each additional monitored region requires intentional actuation of a corresponding muscle group and, in some cases, simultaneous actuation [64]. When used by children, this complexity may be overwhelming and has led to a reported impact on rejection of devices [33].

This team’s unique 3D-printed prosthesis leverages a single EMG measurement, which may support simplicity in daily calibration and application. On-board signal processing can correlate the intensity of the measured muscle contraction or the number of contractions in a specific time period. This can result in actuating different types of hand-state gestures, including individual finger actuation motor position. The limitations associated with current EMG devices are an opportunity to examine improvements to both the design and training methodology.

### 3.4. Control-Gamification and Training

Single-surface EMG providing multigestural control allows the user, using contraction intensity and patterns, to control their prosthesis. Due to the complexity of the controls, a custom video game-based training system was developed to train the user in a risk-free environment. The video game system collects the filtered EMG input from the user and routes it to the computer system; where it is either interpreted as a multifunction controller or analog input. Training systems with mechanics that are similar to the active arm control, similar to punching or slapping, using an EMG controller have increased usability scores [65].

This research team’s newest game for the prosthetic arm training, called Magical Savior of Friends (MSOF), places the character in a ‘Mario style’ side-scrolling game with a magical character that can initiate superpower attacks and defenses based on the amplitude of their contraction. After going through a thresholding calibration sequence, the player is able to vary their measured contraction magnitude, which correlates to how hard the player activates their muscle contraction. For example, calibration can be set for low, mid and max thresholds. Calibration for setting the low threshold is applied to move the reading above the noise threshold. In application, these gestures initiate three completely different superpower attacks to occur.

While the game is designed to be fun and approachable on the surface, it provides meaningful training through simulation to learn complex multigesture hand states. Preliminary results are promising, as players become significantly more accurate with the tuned contraction after playing the game for one hour [66]. By gamifying the training, this method provides many opportunities for failure and feedback in a safe low-pressure environment. Multiple studies have found that feedback is crucial in improving control of a myoelectric prosthesis [33,44]. Disguised as a game, simulation is effective in allowing practice and training the prosthesis user. An extension of this work is underway to provide prosthesis users with new opportunities to play games they otherwise might not have the dexterity or proper interface for.

### 3.5. Comfort and Durability

3D printing, as with most manufacturing processes, has its advantages and limitations. The promise of personalized medicine and ability to rapidly produce models has been shown to be effective in the clinical setting [67]. While not significantly impacting professionals that use 3D printing to help to plan procedures via the production of representative models, concerns for the safety of 3D-printed parts have been expressed [68]. Professionals using material properties to optimize design must be aware of the manufacturing process’ impact. In testing of additive manufacturing ABS plastic, samples have been reported to have variable mechanical properties based on print orientation and are estimated to have between 10% to 73% of the strength of samples produced by injection molding [69,70]. While the method through which layers are printed has an impact on the deviation from material standards, optimization can be used to improve reliability and predictability of performance [69,71]. In an effort to have consistent and reliable 3D-printed components for use in the medical environment, consistent standards and best practices should be implemented. While FDA guidelines make many recommendations toward work-flow and documentation for part tracking in the event of a part failure [71], better understanding the underlying principles behind part vulnerability can help to minimize those risks preemptively. When designed with realistic loading expectations and considerations for manufacturing, additive manufacturing can provide stable and resilient parts that can reduce overall system weight and manufacturing costs [72].

## 4. Discussion of Regulatory Framework

While a tremendous body of work is available on attempts to advance the novel manufacturing techniques, reviews of various studies have identified areas for continued work [73,74]. An independent review [73] of 314 current studies evaluated for levels of evidence and validity highlighted a trend of the reports to be more in line with case studies as opposed to randomized control trials. Several areas of evidence and clarity, including sufficient study power, statistical tests, reliable outcome measures, and clarity in recruitment, were all identified as needing expounding. The review [73] called for a significant appraisal of both efficacy and effectiveness to provide healthcare professionals with more information to make critical decisions on readiness for broader patient care.

In order to advance the state-of-the-art and to quantify the impact and effectiveness of our research team’s new 3D-printed electromyographically actuated multigesture arms, a novel clinical trial has been proposed in collaboration between Oregon Health & Science University and the University of Central Florida. This study is considered nonsignificant-risk. Twenty patients between the ages of 6 and 17 will participate in a one-year clinical trial with four total assessments. Assessment has been designed in two parts: Influence on quality of life (Children’s Hand-Use Experience Questionnaire (CHEQ) and PedsQL) and myoelectric control (Assessment of capacity for Myoelectric Control (ACMC)).

CHEQ uses a four-category rating scale to assess the functionality and limitations of a child, developed for ages 6 through 18 years old, and is available on the internet (www.cheq.se) for easy access [75]. It uses a variety of questions with a nested structure, such as: “The first question reads: ‘Is this something you usually do independently?’ and has the response options:

- ‘yes’
- ‘no’
- ‘I get help/avoid doing it’
- or ‘not applicable’.

If the answer is ‘no’ or ‘not applicable’, the item is scored as missing, and the respondent moves to the next item. If the answer is ‘yes’, the second opening question appears: ‘Do you use one hand or both hands together?’, with the response options:

- ‘one hand’
- ‘both hands’
- ‘with the involved hand supporting but not holding’
- ‘both hands, with the involved hand holding the object’.

This type of assessment [75] provides a reliable baseline to understand how the child’s daily life is influenced by their limb difference.

PedsQL is a well-validated survey that asks twenty-three questions of both parents and children about various aspects of health-related quality of life over the past month. Published results are available for the general population. The PedsQL scoring algorithm translates the available responses to questions (“never”, “almost never”, “sometimes”, “often”, or “almost always”) into scores of 0%, 25%, 50%, 75%, and a maximum of 100% for each of the four generic core scales (Physical Health, Emotional Functioning, Social Functioning, and School Functioning).

Assessment of capacity for myoelectric control (ACMC) [76,77] is a Rasch rating scale that is used to detect expected change in a person’s ability using objective variables. There are 30 items that evaluate a prosthetic arm’s ability to do specific functions that involve gripping, holding, releasing, and coordinating between limbs. This is done by asking the subject to perform certain tasks and scoring their motions, including traditional chores such as making simple meals and setting a table. Additional questions look at hobby and leisure activities, such as assembling a simple project such as LEGO bricks [76,77]. The occupational therapy team supporting the evaluation will support evaluation of performance, with feedback from the study participants documented. The findings from this study will be reported following the assessment and evaluation, and the data used to continue to improve both the design methodology and study methodology.

## 5. Conclusions

The outlook for using 3D printing manufacturing techniques and collaborative design is bright, with rapidly progressing iteration and designs that can better develop affinities for users. At this time, limited work has been reported involving sufficient power and clinical assessment [73,74]. By designing and conducting novel clinical assessment of these electromyographic 3D printed bionic limbs with well defined outcome metrics, this may lead to being able to add to the field and better capture the readiness for broader distribution. Continuing efforts to validate and assess both design and performance will improve translation of the technology and design methods. The process for designing specifically for the end=user with significant reduction in costs may radically change the accessibility of functional prosthesis for pediatric patients.
